# Implementation and validation of a 24/7 system for the monitoring of antiepileptic drugs

**DOI:** 10.3389/fneur.2025.1493201

**Published:** 2025-03-14

**Authors:** Tatjana Khromov, Gry Helene Dihazi, Phillipp Brockmeyer, Andreas Fischer, Frank Streit

**Affiliations:** ^1^Department of Clinical Chemistry, University Medical Center Goettingen, Goettingen, Germany; ^2^Department of Oral and Maxillofacial Surgery, University Medical Center Goettingen, Goettingen, Germany

**Keywords:** epilepsy, therapeutic drug monitoring, 24/7 automation, antiepileptic drugs, LC-MS/MS, CLAM-2030

## Abstract

**Background:**

Epilepsy is a common neurological disorder associated with seizures that impact patients' quality of life. Treatment includes antiepileptic drugs (AEDs), each effective only at a specific dose, making continuous therapeutic drug monitoring (TDM) useful in clinical cases under inpatient conditions. Conventional liquid chromatography-tandem mass spectrometry (LC-MS/MS) lacks automation for 24/7 operation, limiting clinical applicability. This study validates a fully automated 24/7 AED monitoring system using the Clinical Laboratory Automated Sample Preparation Module 2030 (CLAM-2030).

**Methods:**

The method was validated according to U.S. Food and Drug Administration (FDA) and European Medicines Agency (EMA) guidelines by evaluating linearity, precision, accuracy, carry over, matrix effects, and calibration stability. Twenty-six AEDs were quantified in plasma using multiple reaction monitoring (MRM) transitions in positive and negative electrospray ionization modes. Sample preparation was fully automated: 20 μL methanol was used to wet the column, followed by 20 μL internal standard and 100 μL acetonitrile for protein precipitation. The supernatant was filtered and injected directly into the LC system. Chromatographic separation was achieved within 4.5 min using a C18 column (2.1 × 50 mm, 2.7 μm) under gradient conditions with a mobile phase of 0.2 mM ammonium formate and 0.002% formic acid.

**Results:**

The method demonstrated excellent linearity over the validated concentration ranges (*R*^2^ > 0.99 for all analytes). Within-run imprecision was <15% at the lower limit of quantitation (LLOQ), while between-run imprecision was <10% for most AEDs. Accuracy was within ±10% of nominal concentrations at all quality control (QC) levels. Matrix effects were within acceptable limits (<30% variation) for 23 of 26 analytes, with compensatory corrections applied for carbamazepine-D_10_, felbamate-D_4_, and levetiracetam-D_6_. Carry over was negligible [<2% for all AEDs except retigabine and N-desmethylselegiline (NDMS), which remained below 6.5%]. Calibration stability was maintained over 5 days with concentration and peak area variation <10%. An interlaboratory comparison (ring test) showed a relative standard deviation <20% for all analytes.

**Conclusion:**

This study establishes a robust, fully automated, high-throughput method for continuous AED monitoring in the clinical setting. The CLAM-2030-LCMS-8060NX system enables reliable 24/7 TDM with minimal technical expertise, ensuring optimized AED therapy and improved patient outcomes.

## 1 Introduction

Epilepsy is a chronic neurological disorder characterized by recurrent seizures that can significantly impair patients' quality of life (1, 2). Seizures, the hallmark symptom of epilepsy, present with diverse manifestations, including muscle spasms, loss of consciousness, and behavioral changes (1). The global prevalence of epilepsy ranges from 4 to 10 cases per 1,000 persons, with variations in different populations (3). Epilepsy can be caused by a variety of different factors, including genetic predisposition, brain injury, infection, and developmental disorders (4, 5).

Pharmacological treatment relies primarily on antiepileptic drugs (AEDs) to suppress seizures and relieve symptoms (6). However, to achieve therapeutic efficacy, AED concentrations must be maintained within a specific range, as even small dose variations can lead to disproportionate changes in plasma levels (7). The optimal dose is determined by balancing clinical efficacy and tolerability, taking into account potential adverse effects (8). AED pharmacokinetics exhibit significant inter-individual variability, particularly in populations with altered drug metabolism, such as children, the elderly, pregnant women, and patients with impaired hepatic clearance (7). In addition, polytherapy is often required in refractory epilepsy, which increases the risk of pharmacokinetic interactions and treatment failure or toxicity (6, 9).

Therapeutic drug monitoring (TDM) plays a critical role in optimizing AED therapy by individualizing dosing regimens based on plasma drug concentrations (10, 11). TDM facilitates dose adjustments, evaluates patient adherence, and minimizes adverse effects (12, 13). Its use is particularly valuable in special populations, such as pregnant women, where maintaining therapeutic drug levels while minimizing teratogenic risks is critical (14), and in patients with hepatic impairment, where altered drug metabolism requires careful dose modification (15). In addition, TDM assists in the evaluation of AED-drug interactions to ensure effective seizure control while reducing toxicity risks (16).

Liquid chromatography coupled to tandem mass spectrometry (LC-MS/MS) is the gold standard for the simultaneous quantification of multiple AEDs, offering high sensitivity and specificity (17). However, conventional LC-MS/MS systems often lack the operational feasibility required for continuous 24/7 monitoring, particularly in laboratories with rotating and less experienced staff (18). The Clinical Laboratory Automated Sample Preparation Module 2030 (CLAM-2030, Shimadzu, Kyoto, Japan), in conjunction with the LCMS-8060NX system, addresses these limitations by enabling fully automated sample preparation and LC-MS analysis, thereby facilitating 24/7 drug level monitoring (19). The system is amongst others equipped with a user-friendly Health Level 7 (HL7) interface and provides an easy way to determine drug levels around the clock through automated sample preparation and LC-MS analysis (19).

This study aimed to develop and validate a routine 24/7 method for the quantification of 26 AEDs in different clinical settings. Key analytical parameters including linearity, analyte recovery, matrix effects, precision and accuracy were systematically evaluated. In addition, the feasibility of implementing this system in a clinical laboratory environment operated by technicians with no prior experience in chromatography or mass spectrometry was evaluated.

## 2 Material and methods

### 2.1 Patient blood samples

Patients' blood samples were collected as part of routine clinical diagnostics. The patients provided written informed consent to participate in this study as part of their treatment contract. The study was reviewed and approved by a local ethics committee (No: 6/4/18).

### 2.2 Chemicals and reagents

The calibrators and quality controls (Recipe, Munich, Germany) used in this study were based on human serum. Commercially lyophilized materials were reconstituted in distilled water, aliquoted to 75 μL, and stored at −20°C. The internal standard was prepared in an albumin oxide solution (lot number MKBJ1604V, Sigma-Aldrich, Buchs, Switzerland) containing 4% albumin in 0.9% NaCl and stored at −20°C in 1 mL aliquots.

### 2.3 Sample preparation

Plasma protein precipitation and filtration were performed fully automatically using the CLAM-2030 system (Shimadzu, Kyoto, Japan) before the filtrate was transferred to the LC system. For sample preparation of calibrators, quality controls (QCs), and patient samples, 20 μL of 100% methanol was first added to wet the column, followed by 20 μL of internal standard and 100 μL of 100% acetonitrile. The mixture was vortexed for 45 s, centrifuged at 1,900 rpm and filtered on the CLAM-2030 for 45 s to separate precipitated proteins from the analytes. The final injection volume was 0.5 μL.

### 2.4 Chromatographic separation

The chromatographic separation of AEDs was achieved using a steep ascending gradient with mobile phase A (H_2_O including 0.2 mM ammonium formate and 0.002% formic acid) and mobile phase B (methanol including 0.2 mM ammonium formate and 0.002% formic acid) at a flow rate of 0.5 mL/min in gradient mode. The gradient started with 1% phase B at 0.5 min, increased sequentially to 9% at 0.7 min, 25% at 1.0 min, 45% at 2.2 min, 65% at 2.6 min, 80% at 2.9 min, and reached 95% at 3.0 min. Phase B was maintained at 95% until 3.5 min, then reduced to 1% at 3.6 min, followed by re-equilibration at 1% until 4.3 min. The total analysis time was 4.5 min. Separation was performed on a C18 column (2.1 × 50 mm, 2.7 μm; Shimadzu, Kyoto, Japan) at 30°C.

### 2.5 Mass spectrometry

Experiments were performed on a triple quadrupole electrospray ionization (ESI) mass spectrometer operating in both positive and negative ion modes. Analytes were fragmented using argon 5.0 as the collision gas, and optimized multiple reaction monitoring (MRM) transitions were recorded for each compound. Quantification was based on the peak area ratios of AEDs to their corresponding isotopically labeled internal standards. MRM transitions and retention times (RT) for the analytes are given in Table 1.

**Table 1:** Multiple reaction monitoring (MRM) transitions and retention times (RT) for all 26 antiepileptic drugs (AEDs).

**Analyte**	**Multiple reaction monitoring (MRM) [m/z]**	**Retention times (RT) [min]**
10-OH-Carbamazepine	255.11 → 141.00	2.7
Carbamazepine	237.10 → 165.05	3.1
Carbamazepine-diol	271.10 → 180.00	2.6
Carbamazepine-epoxide	253.09 → 180.05	2.7
Ethosuximide	140.07 → 42.00	1.8
Felbamate	239.10 → 178.05	2.3
Gabapentin	172.13 → 55.00	1.2
Lacosamide	251.14 → 91.10	2.2
Lamotrigine	256.00 → 58.10	1.8
Levetiracetam	171.11 → 126.00	1.7
N-Dimethylsulfoxide (NDMS)	188.07 → 188.07	2.6
Oxcarbazepine	253.09 → 180.00	2.9
Phenylethylmalonamide (PEMA)	207.11 → 91.10	1.7
Perampanel	350.13 → 219.05	3.3
Phenobarbital	231.08 → 42.00	2.6
Phenytoin	251.08 → 102.10	3.1
Pregabalin	160.13 → 97.20	1.1
Primidone	219.11 → 162.00	2.3
Retigabine	304.15 → 230.05	3.2
Rufinamide	240.07 → 127.00	2.2
Stiripentol	217.12 → 145.15	3.5
Sultiame	289.03 → 225.00	1.8
Tiagabine	376.14 → 247.05	3.3
Topiramate	338.09 → 78.00	2.7
Valproate	143.10 → 143.10	3.4
Zonisamide	211.02 → 118.00	1.9

## 3 Results

To validate the multiparameter method for 26 AEDs, a comprehensive validation was performed in accordance with U.S. Food and Drug Administration (FDA) and European Medicines Agency (EMA) guidelines. The process included assessment of linearity, precision, imprecision, calibration stability, exclusion of matrix effects, and carry over testing. All measurements were performed using the fully automated CLAM-2030-LCMS-8060NX system and analyzed using LabSolution LCMS Ver. 5.109 software (Shimadzu, Kyoto, Japan).

### 3.1 Validation of high-performance liquid chromatography (HPLC) assays

**Figure 1:**
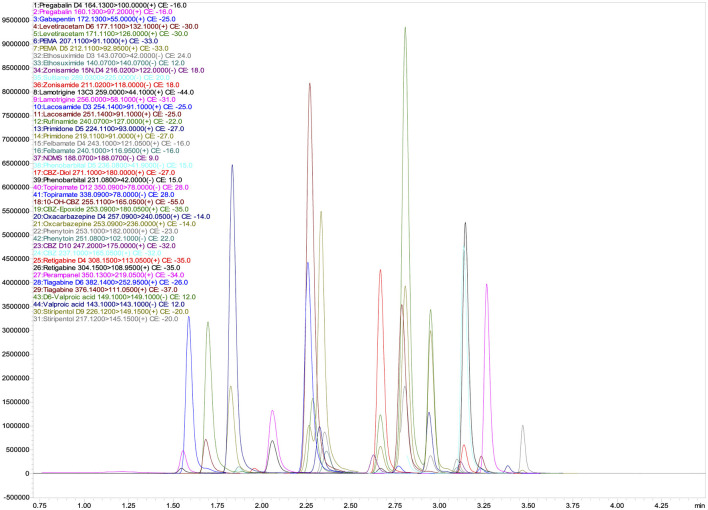
UHPLC-MS/MS chromatograms of quantifier ions for 26 Antiepileptic Drugs (AEDs) and their 17 isotopically labeled internal standards (ISs).

The total run time for the analysis of all analytes was 4.5 min, ensuring high throughput performance and efficiency. Figure 1 shows the corresponding chromatographic separation, highlighting the retention times and peak resolution of the quantifier. The optimized method provides reliable and reproducible results, essential for accurate TDM in clinical applications.

### 3.2 Selectivity and specificity studies

In addition to monitoring the MRM transitions of the quantifier and qualifier, we evaluated the selectivity of the LC-MS system coupled to upstream UHPLC by chromatographic separation. No significant interferences were detected in our measurements, confirming the specificity of the method for accurate quantification of the target analytes.

### 3.3 Imprecision and accuracy

The imprecision and accuracy of the method were evaluated using samples of five different concentrations—Quality Control (QC) levels 1 and 2 and Calibrators (Cal) 1, 2, and 3—for all AEDs tested in both within-run (intra-batch) and between-run (inter-batch, *n* = 5) measurements. Table 2 presents the results of the within-run analysis, while Table 3 summarizes the between-run performance for a selection of the most commonly used AEDs in clinical practice (20). The full dataset for all 26 AEDs is available online at https://doi.org/10.25625/NTQNU5.

**Table 2:** Within-run analytical accuracy and imprecision of the five most commonly used antiepileptic drugs (AEDs) in clinical practice.

**Sample**	**Valproate**					**Carbamazepine**					**Phenytoin**					**Phenobarbital**					**Levetiracetam**				
	**QC1**	**QC2**	**Cal1**	**Cal2**	**Cal3**	**QC1**	**QC2**	**Cal1**	**Cal2**	**Cal3**	**QC1**	**QC2**	**Cal1**	**Cal2**	**Cal3**	**QC1**	**QC2**	**Cal1**	**Cal2**	**Cal3**	**QC1**	**QC2**	**Cal1**	**Cal2**	**Cal3**
Concentration target value [μg/ml]	22.9	51.8	2.2	37.9	112.0	18.6	42.0	6.3	33.7	91.2	5.1	12.6	1.7	8.7	27.5	9.2	22.0	3.7	17.2	53.1	12.3	28.6	3.9	21.6	63.1
Concentration mean value [μg/ml]	24.2	54.6	2.2	39.7	117.6	18.7	42.9	5.5	34.9	89.8	5.9	13.8	1.6	8.4	27.9	10.3	23.2	3.3	18.5	54.6	12.5	28.9	3.9	21.6	60.0
Bias d (%)	5.5	5.3	−0.1	4.7	5.0	0.8	2.1	−12.7	3.6	−1.5	16.6	9.4	−5.3	−3.3	1.4	11.2	5.6	−12.6	7.6	2.8	1.7	0.9	−1.0	−0.2	−5.0
Imprecision CV (%)	2.5	2.4	12.6	6.4	2.5	3.8	2.3	5.8	3.8	1.7	2.8	2.8	5.5	3.4	2.4	2.1	3.4	5.3	3.9	1.7	4.7	2.6	3.4	2.5	6.9

Given their narrow therapeutic index, dose-dependent toxicity, or complex pharmacokinetics, regular therapeutic drug monitoring (TDM) of serum levels is clinically essential.

**Table 3:** Between-run analytical accuracy and imprecision of the five most commonly used antiepileptic drugs (AEDs) in clinical practice over 5 days.

**Sample**	**Valproate**					**Carbamazepine**					**Phenytoin**					**Phenobarbital**					**Levetiracetam**				
	**QC1**	**QC2**	**Cal1**	**Cal2**	**Cal3**	**QC1**	**QC2**	**Cal1**	**Cal2**	**Cal3**	**QC1**	**QC2**	**Cal1**	**Cal2**	**Cal3**	**QC1**	**QC2**	**Cal1**	**Cal2**	**Cal3**	**QC1**	**QC2**	**Cal1**	**Cal2**	**Cal3**
Concentration target value [μg/ml]	22.9	51.8	8.6	38.1	112.0	18.6	42.0	6.2	32.0	90.2	5.1	12.6	1.9	9.0	28.9	9.2	22.0	3.6	18.0	53.7	12.3	28.6	4.2	21.9	67.1
Concentration mean value [μg/ml]	23.5	52.8	8.2	39.4	113.2	18.8	42.0	6.8	32.5	89.1	5.7	13.2	2.0	9.9	29.4	10.0	23.5	3.6	18.5	53.6	12.4	28.8	4.6	22.4	67.2
Bias d (%)	2.8	1.9	−4.0	1.5	1.1	0.9	0.0	10.8	1.7	−1.2	12.9	9.7	2.0	10.0	1.7	8.2	6.9	0.5	2.9	−0.2	1.1	0.6	8.5	2.2	0.1
Imprecision CV (%)	4.8	4.5	6.7	3.9	3.1	3.5	2.2	2.9	2.7	2.5	6.1	5.7	9.3	7.9	5.8	4.6	3.5	4.8	5.0	2.0	3.8	2.4	2.2	4.3	4.7

Due to their narrow therapeutic index, dose-dependent toxicity, or complex pharmacokinetics, regular therapeutic drug monitoring (TDM) of serum levels is clinically essential.

The within-run imprecision for the 26 AEDs was generally low, with coefficients of variation (CVs) below 15% for all analytes, acceptance criteria. The LLOQ imprecision was also <15%, meeting EMA and FDA requirements. However, all values remained within acceptable limits, ensuring the reliability of the method for routine clinical use.

The between-run imprecision showed consistent performance, with CV values predominantly below 10% for most analytes, indicating high reproducibility over several days.

### 3.4 Linearity

The linearity of the method was evaluated using the highest calibrator (Cal3) and its five serial dilutions. The validated linear range included the known therapeutic concentrations of the drugs as well as their laboratory alert levels (21). Samples exceeding the upper limit of the linear range can be automatically diluted 1:2, 1:5, or 1:10 by the instrument in cases of suspected intoxication. Table 4 shows the calibration regression function (Y), correlation coefficient (*R*^2^) and *P*-values from simple regression analysis for a selection of the most commonly used AEDs in clinical practice (20). The full dataset for all 26 AEDs is available online at https://doi.org/10.25625/NTQNU5.

**Table 4:** Linearity, therapeutic range and laboratory alert values of the five most commonly used antiepileptic drugs (AEDs) in clinical practice.

**Analyte**	**Y**	***R* ^2^**	***P*-value**	**Range** **[μg/ml]**	**Therapeutic range [μg/ml]**	**Laboratory alert level [μg/ml]**
Valproate	1.0656 × +0.0309	0.9963	<0.0001	3.5–112	50–100	120
Carbamazepine	0.9832 × +1.2293	0.9987	<0.0001	2.85–91.2	4–12^*^	20
Phenytoin	1.02 × −0.303	0.9998	<0.0001	0.9–27.5	10–20	25
Phenobarbital	1.0326 × −0.4976	0.9999	<0.0001	1.7–53.1	10–40	50
Levetiracetam	0.9631 × −0.0464	0.9985	<0.0001	2–63.1	10–40	50

* The reference range refers to the sum of carbamazepine and carbamazepine epoxide (active metabolite of carbamazepine). Statistical test, simple linear regression (*n* = 6).

The study confirms that all AEDs fall within their established therapeutic windows, supporting their applicability for TDM. The method showed excellent linearity over the entire concentration range (*R*^2^ > 0.99). Calibrators and controls adequately cover the therapeutic range, ensuring accurate quantification. In addition, the measurement range extends to intoxication levels, primarily due to the instrument's automatic dilution capability (1:2, 1:5, 1:10), further enhancing the method's suitability for clinical use.

### 3.5 Matrix effects

To exclude potential matrix effects, we performed several experiments. First, we analyzed the ratio of isotopically labeled internal standard area in a blank matrix (0.9% NaCl) compared to those in Cal3 (based on human serum). For most internal standards, this ratio remained below 30%, indicating minimal matrix interference. However, a matrix effect was observed for carbamazepine-D_10_, felbamate-D_4_ and levetiracetam-D_6_ where the ratio exceeded 30%. In addition, we performed dilution experiments using Cal2 diluted in three different patient samples at three different ratios (3:1, 1:1, 1:3) according to EMA guidelines. The observed inaccuracy remained within 20%, suggesting effective compensation of matrix effects by the internal standard (data not shown).

### 3.6 Carry over

Carry over was assessed by measuring a blank sample immediately after Cal3 (the highest concentration) to ensure that the residual signal did not exceed 20% of the limit of detection (LOD), which in this case corresponds to Cal1. The analysis confirmed that the carry over in the blank was well below this threshold [carryover = (MV blank/MV LLOQ) × 100]. For 26 AEDs, carry over effects were minimal, with most analytes showing values below 2%. However, N-desmethylselegiline (NDMS, 6.35%) and retigabine (6.12%) showed the highest carry over, indicating the need for additional precautions, such as extended system rinses, to prevent cross-contamination in subsequent analyses.

### 3.7 Calibration stability

Between-run precision and imprecision were assessed by analyzing aliquots of patient and quality control samples over five consecutive days using a single calibration curve established on day 1. Calibration stability was confirmed as the imprecision of both concentration and peak area for quality control and patient samples remained within 10%, meeting EMA acceptance criteria.

### 3.8 Interlaboratory (ring trial) analysis

To assess the suitability of the method, we participated in two collaborative trials conducted 6 months apart, analyzing a total of four interlaboratory test samples to evaluate concentration accuracy. The relative standard deviation remained below 20% (data not shown), demonstrating the reliability of the method in an interlaboratory setting.

## 4 Discussion

Epilepsy is a chronic neurological disorder that affects approximately 1% of the world's population and requires long-term treatment with AEDs to control seizures and improve quality of life (22, 23). However, AED therapy presents significant challenges, including drug resistance, interindividual pharmacokinetic variability, and potential adverse effects, particularly in critically ill patients (24). These complexities underscore the need for personalized treatment strategies to optimize therapeutic outcomes (25).

TDM is essential to ensure that AED concentrations remain within the therapeutic range, thereby maximizing efficacy while minimizing toxicity (24). Individual patient factors, such as genetic polymorphisms, drug metabolism, and pharmacokinetic interactions, significantly influence AED levels and require accurate and timely monitoring (26). Although not yet standard in routine clinical practice, TDM has demonstrated benefits in improving dosing accuracy, enhancing therapeutic efficacy, and reducing adverse effects, highlighting its clinical relevance (27, 28).

Traditional TDM relies on intermittent blood sampling, which may not adequately capture fluctuations in drug concentrations. In contrast, a fully automated 24/7 TDM system allows continuous real-time monitoring, providing a dynamic and personalized approach to AED therapy (29). Given the narrow therapeutic index of many AEDs, stable plasma concentrations are critical for effective seizure control, making real-time monitoring particularly valuable (30). Integrating 24/7 TDM into clinical workflows offers several advantages, including proactive dose adjustments based on real-time data, improved safety in acute settings such as status epilepticus, and improved adherence through early identification of non-compliance (31, 32).

LC-MS/MS is widely considered the gold standard for TDM due to its high specificity, precision, and sensitivity (33). However, conventional LC-MS/MS methods require labor-intensive sample preparation, specialized personnel, and extensive training, limiting their accessibility in routine clinical practice. To address these limitations, this study developed and validated a fully automated 24/7 LC-MS/MS-based TDM system using the CLAM-2030 platform. This system automates sample preparation, minimizes manual handling, and enables high-throughput AED quantification, making it feasible for use by laboratorians without prior chromatography or mass spectrometry expertise.

Extensive validation of the system confirmed its analytical robustness. Key performance parameters included isobaric resolution (>1), calibration accuracy (80–120%) over seven days, repeatability (CV <10%), intra-day precision (CV <15%), matrix effect (matrix factor 50–120%), and carry over elimination (blank-to-LLOQ ratio <30%). In addition, interlaboratory validation demonstrated accuracy within 80–120%, supporting the clinical applicability and reproducibility of the system. The feasibility of routine 24/7 AED monitoring was successfully demonstrated, highlighting the potential for widespread implementation in clinical laboratories.

Despite its advantages, fully automated 24/7 TDM faces several challenges (34). Technological limitations may affect the system's ability to accurately quantify AEDs at very low plasma concentrations or in complex biological matrices, requiring continuous improvements in sensitivity and specificity (35). In addition, inter-individual variability due to genetic polymorphisms, age, and comorbidities may complicate the interpretation of TDM results, highlighting the need for further personalization of treatment strategies (36). Economic considerations are also a barrier, as the cost of implementing and maintaining automated TDM systems may limit accessibility, particularly in resource-constrained settings (37). Cost-benefit analyses will be essential to facilitate wider adoption.

Future advances in automated TDM should focus on the integration of pharmacogenetic data to enable truly personalized AED therapy by accounting for genetic variations in drug metabolism (38). In addition, the development of point-of-care TDM devices could improve real-time drug monitoring, allowing for immediate dose adjustments and improved patient outcomes (39). Expansion of the system to include newer-generation AEDs will be necessary to keep pace with advances in epilepsy treatment and ensure comprehensive drug monitoring (40).

By addressing these challenges and advancing TDM technology, fully automated 24/7 monitoring has the potential to transform epilepsy management by enabling more precise, timely, and individualized treatment strategies. This study successfully demonstrated the feasibility of continuous AED monitoring using an automated LC-MS/MS platform, reducing reliance on specialized personnel while maintaining high analytical performance. As the treatment of epilepsy continues to evolve, the integration of real-time TDM into routine clinical practice represents a significant step toward optimizing therapeutic outcomes and improving patient care.

## 5 Conclusions

This method offers several advantages over conventional LC-MS methods. Key benefits include superior calibration stability (eliminating the need for daily calibration), ease-of-use with minimal technical expertise, and automated sample preparation that reduces the time from sample receipt to result output to <10 min. In addition, the system allows seamless switching between analytical methods without rinsing or equilibration, supports analysis of samples in any order of arrival (including those requiring different methods), and provides seamless LIS integration via an HL7 interface. The exceptional robustness of the instrument allows uninterrupted 24/7 operation.

## Data Availability

The raw data supporting the conclusions of this article will be made available by the authors, without undue reservation.
